# Evaluation of pharmacist consults within a collaborative enhanced primary care team model to improve diabetes care

**DOI:** 10.1371/journal.pone.0280654

**Published:** 2023-01-20

**Authors:** Danielle Firkus, Rozalina G. McCoy, John Matulis, Maya Kessler, Kristin Mara, Joseph Herges

**Affiliations:** 1 Department of Pharmacy, Mayo Clinic, Rochester, Minnesota, United States of America; 2 Division of Community Internal Medicine, Geriatrics, and Palliative Care, Department of Medicine, Mayo Clinic, Rochester, Minnesota, United States of America; 3 Division of Health Care Delivery Research, Mayo Clinic Robert D. and Patricia E. Kern Center for the Science of Health Care Delivery, Rochester, Minnesota, United States of America; 4 Division of Biomedical Statistics and Informatics, Mayo Clinic, Rochester, Minnesota, United States of America; University of Verona, ITALY

## Abstract

**Background:**

An enhanced primary care team model was implemented to provide proactive, longitudinal care to patients with diabetes, grounded in close partnership between primary care providers (PCPs), nurses, and Medication Management Services (MMS) pharmacists. The purpose of this study is to evaluate the impact of the MMS pharmacist involvement in the enhanced primary care model for patients with diabetes.

**Methods:**

This retrospective cohort study compared the quality of diabetes care between patients referred to a pharmacist and propensity score matched controls who were not. Eligible patients were adults (age 18 to 75 years) enrolled in the enhanced primary care team process who did not meet at least one of four diabetes quality indicators at 13 Mayo Clinic Rochester primary care practice locations. The intervention examined was asynchronous e-consults by pharmacists affiliated with the primary care practice.

**Main measures:**

The primary outcome was change in the proportion of patients meeting the composite of four diabetes treatment goals (D4), including hemoglobin A1c (HbA1c) control, blood pressure control, aspirin use, and statin use at six months from enrollment among patients who received pharmacist intervention compared to matched patients who did not. Secondary outcomes were each of the D4 goal individually.

**Results:**

The proportion of patients meeting the D4 increased with pharmacist e-consults (N = 85) compared to matched controls with no review (N = 170) (27% vs 7.0%, p<0.001). The change in patients meeting treatment goals of HbA1c (12.9% vs 4.1%, p = 0.020), blood pressure (9.4% vs 2.4%, p = 0.023), aspirin use (10.6% vs 2.9%, p = 0.018), and statin use (17.6% vs -1.2%, p<0.001) all increased with pharmacist e-consults.

**Conclusions:**

Pharmacist engagement in the enhanced primary care team improved diabetes management. This supports the inclusion and utilization of pharmacists in multidisciplinary efforts to improve diabetes care.

## Introduction

Diabetes is one of the leading causes of morbidity and death in the United States, affecting 37.3 million Americans in 2019 [1]. Timely and evidence-based control of hyperglycemia and cardiovascular disease risk factors (i.e. hypertension, hyperlipidemia, and tobacco use) is critical for reducing the impact of diabetes on patient health and quality of life [2]. Despite advances in the science of diabetes management, control of these risk factors remains poor [3–5]. To help clinicians better care for people living with diabetes, the American Diabetes Association endorses the use of collaborative practice models to improve adherence to recommended standards of care [6]. Collaborative models leverage the expertise and unique skill sets of various care team members to enhance patient care [7].

To improve the care provided for this complex and multifaceted condition and to address the increasing demand for health services stemming from increased prevalence of diabetes and comorbidity and a diminishing primary care workforce, innovative care models utilizing pharmacist consultative services offer a promising optimization of patient care [8]. While all pharmacists now receive a Doctor of Pharmacy degree, which includes extensive training in pharmacology, physiology, and pathophysiology, many also undergo additional postgraduate residency training. Postgraduate residency training specific to ambulatory care allows a pharmacist to specialize in outpatient chronic disease management. The residency provides a structured clinical experience with a mentor that facilitates the development of Doctor of Pharmacy graduates into independently functioning practitioners. Medication Management Services (MMS) pharmacists practice in outpatient care settings, including in primary care with implementation of collaborative practice agreements with physicians, and can initiate, modify, and discontinue medications and order laboratory tests as clinically necessary for drug therapy monitoring. As a result, pharmacists can work with patients and other members of the primary care team to optimize patient outcomes through medication management, coordination of care, and patient education [9]. Pharmacist engagement has been shown to enhance adherence to prescribed glucose-lowering medications and improve HbA1c, blood pressure, and lipid profiles. The most common way for pharmacists to be incorporated into collaborative models of diabetes care is as part of a consultative service outside of the construct of the primary care team [10]. Within such models, pharmacists see patients for scheduled appointments, which—while effective—may not always be feasible given constraints on the healthcare system and lack of available pharmacists at all locations or in sufficient numbers to accommodate the need for their services.

We have previously reported the development and implementation of an enhanced primary care team model comprised of care team registered nurses (RNs) and MMS pharmacists designed to improve the quality of diabetes care provided to patients [11]. The MMS pharmacist role in this model includes advising the team (RNs and primary care providers (PCP)) through asynchronous patient chart reviews and providing detailed recommendations for diabetes treatment plans, typically without direct patient contact. While previously described pharmacist interventions are primarily obtained through formal referrals or informal contacts by PCPs, this model empowered care team RNs to activate pharmacist services independently and for pharmacists to provide asynchronous recommendations. This allows pharmacists, a limited care team resource, to utilize their comprehensive medication expertise to impact the care of more patients who may not require a formal consult.

While efficient and likely effective in principle, this approach to interdisciplinary diabetes care management has not been previously evaluated for feasibility or effectiveness. Thus, to address this knowledge gap, we examined the feasibility and impact of pharmacist engagement on diabetes care quality, successfully hypothesizing that utilizing the MMS pharmacist team for treatment recommendations would improve treatment goals of diabetes care. Determining the utility of this novel engagement between pharmacists and the care team may help inform best practices for this process and the development of other multidisciplinary models of diabetes and other chronic disease management.

## Methods

### Study design

This retrospective cohort study compared outcomes among adult patients with diabetes whose charts were asynchronously reviewed through an e-consult by a MMS pharmacist as part of the enhanced primary care team diabetes management model, and for whom care recommendations were provided, with outcomes among matched patients with diabetes who were also managed by the enhanced primary care team model but did not receive a pharmacist review. The study was classified as quality improvement and deemed exempt by the Mayo Clinic Institutional Review Board. All included patients provided authorization to use data from the electronic health record (EHR).

### Setting

Mayo Clinic is an integrated healthcare delivery system based in Rochester, MN, U.S.A that delivers the full continuum of care to local, regional, national, and international patients. Thirteen primary care practices located in Olmsted (12) and Dodge (1) counties deliver comprehensive primary care to local patients, Mayo Clinic employees, and their dependents. The practices are located in urban, suburban, and rural areas with both Family Medicine and Internal Medicine practices. All patients are paneled to a staff physician, nurse practitioner (NPs), physician assistant (PAs), resident, or fellow.

### Diabetes treatment goals

A commonly used indicator of diabetes care quality is the D5, developed and used by the Minnesota Community Measurement Program (MNCM), an organization that delivers data to healthcare payer and provider members to illustrate performance on quality and cost measures [12]. The D5 is a set of five treatment goals that, when achieved together, indicate optimal mitigation of preventable future adverse events and high-quality diabetes care. The goals consist of blood pressure (BP) control (BP less than 140/90 mmHg), lowering cholesterol (statin use as indicated based on the patient’s age, low density lipoprotein cholesterol (LDL-C) level, and cardiovascular disease history), glycemic control (HbA1c less than 8%), tobacco-free status, and aspirin use for secondary prevention of cardiovascular disease. MNCM D5 reporting is mandatory for all healthcare practices in the state. These measures are also reported publicly and used for value-based purchasing contracts. In May 2021, Minnesota had an average statewide D5 rating (i.e. 100% compliance, compliance on 5 out of 5 measures) of 45% for all reporting clinics, demonstrating the need for innovative practice models to improve care [13].

### Study population

The study population was comprised of patients meeting eligibility criteria for MNCM D5 reporting. Eligible patients were adults aged ≥18 to ≤75 years old with a diagnosis of type 1 or type 2 diabetes who are paneled to a PCP at one of the Mayo Clinic Rochester primary care clinic locations. Patients were excluded from the study if they were incarcerated, pregnant, enrolled in hospice or receiving palliative care, a permanent nursing home resident, died during the measurement period, declined research authorization, or had not had a PCP visit during the measurement period. Of patients enrolled in the enhanced diabetes care process not meeting D5 treatment goals, those with a MMS pharmacist e-consult and care recommendations documented in the EHR during our study period were compared with those participants without documented pharmacist involvement. Patients who entered the care model between May 2019 and February 2020 were included.

### Intervention

**Fig 1:**
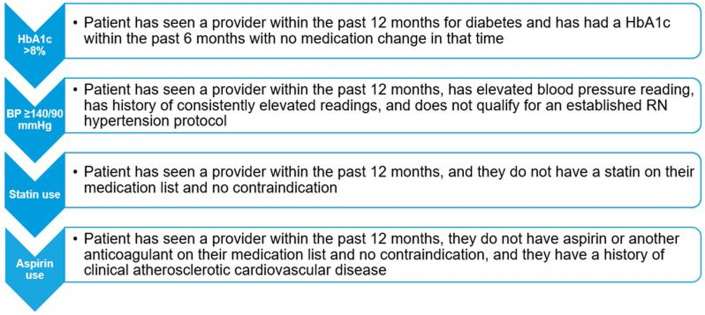
Criteria for pharmacist referral within enhanced primary care team model based on the D4 treatment goals.

The enhanced primary care team model was implemented across all 13 primary care practices of Mayo Clinic Rochester in May 2019 [11]. Briefly, each PCP is paired with a single care team RN; however, a single RN could be partnered with several PCPs depending on the number of paneled patients with diabetes each provider cares for. The RN uses an EHR report to identify patients not meeting the D5 goals. The RN reviewed the EHR, utilized by all providers, to identify and address any outstanding care processes (e.g. measurements of HbA1c, blood pressure, cholesterol, etc.) and used a structured process workflow to solicit assistance from other members of the care team, including a one-time MMS pharmacist e-consult for medication questions and treatment recommendations (Fig 1). The algorithm does not include an option for a formal pharmacist consultation, which would be placed by the PCP and encouraged for more complex patients, such as those on multi-dose insulin regimens or where extensive medical decision making is required. In the EHR message sent to the pharmacist, the RN indicates–using a standard communication note template–specific components of the patient’s care that require the pharmacist’s attention. The pharmacist then reviews the patient’s chart and sends recommendations back to the RN using a standardized note template. Subsequently, the RN discusses this information with the PCP, either in person or via the EHR, to finalize the care plan. Once the care plan is finalized, the RN contacts the patient to assess the current state of diabetes management, treatment adherence, and barriers to care. The RN and the patient then engage in shared decision making, grounded in the care plan formulated by the care team and adapted to the patient’s situation, goals, and preferences. The RN continues to engage with the patient electronically or by telephone on an individualized schedule, seeking pharmacist and/or PCP input as needed to help the patient achieve their diabetes care goals. The PCPs and RNs meet monthly to review panel progress and discuss challenging situations, engaging the pharmacist as needed for further guidance on treatment decisions.

### Outcomes

Our primary outcome was the change in the proportion of patients meeting the D4 treatment goals, which is comprised of HbA1c <8.0%, blood pressure <140/90mmHg, aspirin use for secondary prevention of ischemic vascular disease, and cholesterol lowering through statin use as recommended, between the intervention and control groups at six months from the index date. The tobacco use component of the D5 was not included due to an EHR error identified during the study period that incorrectly reported tobacco use. Our secondary outcomes included change in the proportion of patients meeting each of the four D4 components at six months from the index date. Additionally, we examined the change in the proportion of patients with type 2 diabetes treated with specific classes of evidence-based medications at four weeks from the index date. The 4-week time point was chosen to more reliably connect any medication change to the e-consultation. These were multi-dose insulin (MDI), sodium-glucose co-transporter 2 inhibitors (SGLT2i), glucagon-like peptide-1 receptor agonists (GLP-1 RA), angiotensin-converting enzyme inhibitors (ACEi), and angiotensin receptor blockers (ARB).

### Statistical analysis

We used propensity score (PS) matching to reduce the imbalance of measured baseline characteristics between patients with and without a pharmacist review in a 1:2 manner. The PS model was designed to capture control patients who should have received a pharmacist review but did not. Parameters included in the matching were age, race, diabetes type, baseline HbA1c, baseline blood pressure, number of provider visits in the past year with a visit diagnosis of diabetes, LDL-C level, endocrinology visits in the past year, and insulin use, and was estimated by a multivariable logistic regression model. Patients were then matched on the logit of the PS (± 0.2 standard deviation of the logit of the PS). Chi-square tests were used to compare categorical data, including individual and composite D4 treatment goals between groups. Among those not meeting given individual goals, Wilcoxon rank sum tests were used to compare HbA1c, systolic blood pressure, and diastolic blood pressure values between groups. All analyses were conducted using SAS version 9.4 software (SAS Institute Inc., Cary, NC).

## Results

Our study compared 85 patients with diabetes enrolled in the enhanced primary care team model who received pharmacist e-consultation to 170 matched patients who did not. Baseline characteristics of the two groups are summarized in Table 1, while baseline rates of achievement of each individual D4 component and the composite D4 indicator are shown in Table 2. At baseline, there were significant differences in the rates of attainment of the aspirin goal (81.2% vs 92.4%, p = 0.008) and composite D4 goals (1.2% vs 12.4%, p = 0.003), with more patients not meeting each of these goals in the group that ultimately received a pharmacist review. Rates of attainment of HbA1c, blood pressure, and statin goals were similar between the two groups.

**Table 1:** Baseline patient characteristics.

	Pharmacist Review (N = 85)	No Pharmacist Review (N = 170)	Total (N = 255)	p value
**Age**, years, Mean (SD)	64.5 (8.4)	62.9 (8.5)	63.4 (8.5)	0.13
**Gender**				0.93
Female	40 (47.1%)	79 (46.5%)	119 (46.7%)	
Male	45 (52.9%)	91 (53.5%)	136 (53.3%)	
**Race**				0.68
White	82 (96.5%)	161 (94.7%)	243 (95.3%)	
Black or African American	1 (1.2%)	5 (2.9%)	6 (2.4%)	
Other	2 (2.4%)	4 (2.4%)	6 (2.4%)	
**Ethnicity**				0.17
Central American	1 (1.2%)	0 (0.0%)	1 (0.4%)	
Hispanic or Latino	1 (1.2%)	2 (1.2%)	3 (1.2%)	
Not Hispanic or Latino	83 (97.6%)	162 (95.3%)	245 (96.1%)	
Unknown/Chose Not to Disclose	0 (0.0%)	6 (3.5%)	6 (2.4%)	
**Diabetes Type**				0.87
1	6 (7.1%)	13 (7.6%)	19 (7.5%)	
2	79 (92.9%)	157 (92.4%)	236 (92.5%)	
**Family Medicine**	41 (48.2%)	93 (54.7%)	134 (52.5%)	0.33
**Internal Medicine**	29 (34.1%)	41 (24.1%)	70 (27.5%)	0.12
**Medical Resident Clinic**	15 (17.6%)	36 (21.2%)	51 (20.0%)	0.51
**Language**				0.78
English	84 (98.8%)	166 (97.6%)	250 (98.0%)	
Arabic	0 (0.0%)	1 (0.6%)	1 (0.4%)	
Cambodian (Khmer)	0 (0.0%)	1 (0.6%)	1 (0.4%)	
Somali	1 (1.2%)	1 (0.6%)	2 (0.8%)	
Spanish	0 (0.0%)	1 (0.6%)	1 (0.4%)	
**Primary care visit for diabetes within 1 year of index date**	67 (78.8%)	132 (77.6%)	199 (78.0%)	0.83
**Visit with endocrinologist within 1 year of index date**	13 (15.3%)	36 (21.2%)	49 (19.2%)	0.26
**Treated with insulin at index date**	20 (23.5%)	37 (21.8%)	57 (22.4%)	0.75

**Table 2:** Patients meeting treatment goals at baseline versus 6 months.

	Pharmacist Review (N = 85)	No Pharmacist Review (N = 170)	p value*
**D4 Treatment Goal**			<0.001
Baseline	1 (1.2%)	21 (12.4%)	
6 months	24 (28.2%)	33 (19.4%)	
Total change	27%	7%	
**HbA1c Goal**			0.02
Baseline	52 (61.2%)	109 (64.1%)	
6 months	63 (74.1%)	116 (68.2%)	
Total change	12.9%	4.1%	
**Blood Pressure Goal**			0.02
Baseline	46 (54.1%)	101 (59.4%)	
6 months	54 (63.5%)	105 (61.8%)	
Total change	9.4%	2.4%	
**Statin Goal**			0.001
Baseline	35 (41.2%)	89 (52.4%)	
6 months	50 (58.8%)	87 (51.2%)	
Total change	17.6%	-1.2%	
**Aspirin Goal**			0.02
Baseline	69 (81.2%)	157 (92.4%)	
6 months	78 (91.8%)	162 (95.3%)	
Total change	10.6%	2.9%	

*p values compare the total change for the composite D4 goals and each individual D4 component for patients with and without pharmacist e-consultation.

**Fig 2:**
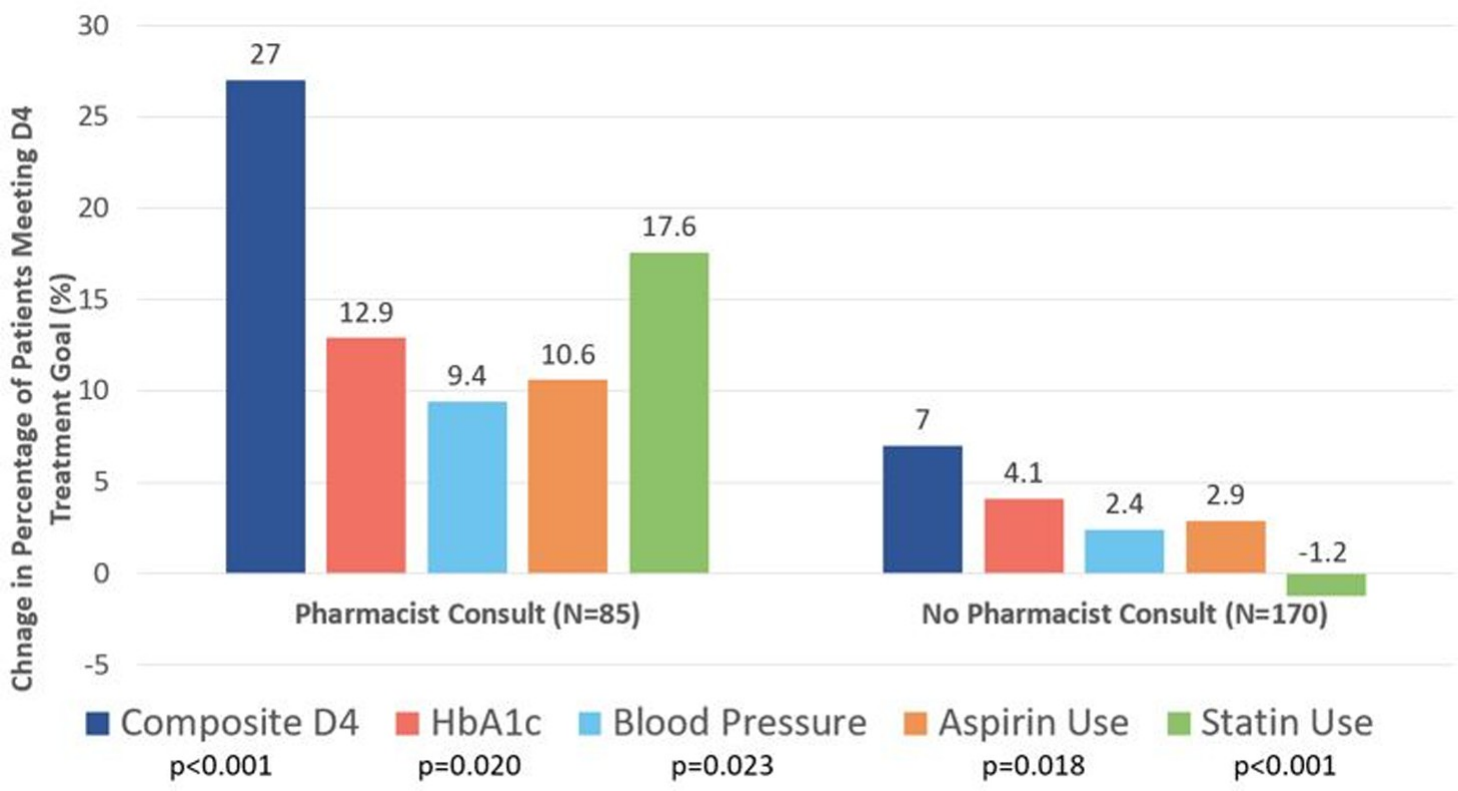
Change in percentage of patients meeting composite and each D4 treatment goal from baseline to 6 months.

The change in the proportion of patients meeting the D4 was greater in the pharmacist intervention group compared to the usual care group after 6 months (27% vs 7%; p<0.001) (Table 2 and Fig 2). The change in the proportion of patients meeting each of the individual D4 treatment goals was also greater in the pharmacist intervention group compared to the usual care group after 6 months, including patients meeting HbA1c goals (12.9% vs 4.1%, p = 0.020), blood pressure goals (9.4% vs 2.4%, p = 0.023), statin goals (17.6% vs -1.2%, p<0.001), and aspirin goals (10.6% vs 2.9%, p = 0.018).

Overall, there were no significant differences between groups in the change from baseline to four weeks from the index date in the proportion of patients taking multi-dose insulin (-1.2% vs 0%, p = 0.70), SGLT2i (0% vs 1.3%, p = 0.81), or ACE-Is/ARBs (2.5% vs 1.2%, p = 0.60). However, there was a larger increase in the percent of patients taking GLP-1 RA from baseline to 4 weeks in the pharmacist review group compared to the non-pharmacist group (3.8% vs 0%, p = 0.035).

## Discussion

In this post-hoc evaluation of an innovative multidisciplinary enhanced primary care model for the management of diabetes implemented across 13 primary care practices in the U.S. Midwest, engaging a pharmacist for treatment recommendations via asynchronous e-consultation significantly improved attainment of diabetes treatment goals over a six-month period. When examined individually, each of the diabetes treatment goals, including HbA1c, blood pressure, aspirin use, and statin use improved with pharmacists’ recommendations on the treatment plan. These data support the integration of pharmacists into the primary care team and demonstrate their ability to efficiently and effectively support chronic disease management through asynchronous e-consultation.

While several prior studies have demonstrated the effectiveness of pharmacist consultations with respect to improving diabetes management, our work extends this benefit to asynchronous e-consultation recommendations organized by primary care nurses, a more efficient alternative to patient consultation that can be scaled and generalized to other settings and populations [10]. In contrast to e-consultations performed in the enhanced primary care team model, in a study by Ip and colleagues, PCPs referred their patients with poor glycemic control to a pharmacist who conducted an initial 45-minute face-to-face patient visit in addition to telephone or face-to-face follow up visits [14]. Although the pharmacist group was significantly more likely to attain goals for HbA1c, LDL-C, and blood pressure reduction, this is a time-consuming process in comparison to the asynchronous e-consultation process utilized in our enhanced primary care team model, which may allow for extended access to pharmacist consultation. A different retrospective study conducted across 53 Veterans Affairs medical centers demonstrated that clinical pharmacy specialists managed the care of ambulatory care patients with hyperglycemia as well as PCPs [15]. In contrast, the enhanced primary care model discussed here extends beyond glycemic control in an effort to more comprehensively mitigate the complications associated with diabetes. Pharmacists can effectively integrate their unique expertise into the daily work of primary care teams, improving outcomes related to blood pressure management, cholesterol lowering, and appropriate aspirin use [16]. With this, pharmacist-led management of diabetes and other cardiovascular disease benefits increases their ability to positively impact patient health outcomes, support PCPs as they care for patients with increasing multimorbidity, and bring their expertise in medication management to a wider range of health conditions requiring treatment intensification.

As primary care practices deliver the majority of care to patients with diabetes across the United States, therapeutic inertia, defined as “a lack of timely adjustment to therapy when a patient’s treatment goals are not met,” is a growing challenge that hinders optimal diabetes management [17]. Comprehensive primary care delivered in accordance with the chronic care model seeks to optimize diabetes management through team-based care, where healthcare providers across disciplines and specialties practice at the top of their skillset and licensure to support optimal diabetes care [7]. Prior models of diabetes care have leveraged nurse practitioners, physician assistants, and clinical pharmacists in this context; however, diabetes care models led by these team members have not been directly compared [18]. The enhanced primary care team model described here illustrates that pharmacists can effectively and efficiently support team-based diabetes care by providing medication recommendations via e-consultation. The backbone of pharmacist training is in pharmacology and pharmaceutical care, which uniquely allows them to assess important medication specific factors, such as drug interactions, dosing with impaired renal/hepatic function, administration considerations, and cost.

The enhanced primary care model illustrates that not all patients with diabetes not meeting care goals require intensive, longitudinal interventions by pharmacists. Pharmacists are a more finite resource than nurses, and the e-consultation process allows pharmacists to improve the care of more patients and for patients located in different physical locations. Longitudinal pharmacist care can then be reserved for more complex patients, such as those on MDI regimens or those not making progress through the enhanced care model. Additionally, pharmacists played a vital role in supporting care team RNs by educating them about potential treatment options to discuss with the PCPs and patients, ultimately empowering and enabling them to take an increasingly active role in patient education and chronic disease management. Pharmacists provide valuable insight and mitigation strategies on medication barriers such as cost, side effects, and administration which can help nurses engage patients in informed shared decision making and ensure that a treatment plan is finalized in one patient conversation, without back-and-forth messaging with the PCP. Pharmacists were also embedded within the primary care clinic with established relationships within the care team, which assists in buy-in to seeking advice and implementing the recommendations. Additionally, this model allows for bi-directional learning between nurses and pharmacists through direct interactions, leading to improved collaboration among primary care team members.

When examining the use of specific medication classes between the groups, we found that only GLP-1RA prescribing increased in the pharmacist e-consult group. A larger sample size may be needed to detect differences in multi-dose insulin or SGLT2i use between groups. The high baseline rate of ACEi/ARB use, nurses and PCPs familiarity with these established medication classes, and an existing EHR prompt to prescribe ACEi/ARB to patients with diabetes and hypertension may explain why these rates did not differ between the two groups.

Strengths of the study include the assessment of multiple clinics in urban, suburban, and rural areas and within both Family Medicine and Internal Medicine practices, which increases generalizability. The pharmacist intervention is generalizable and feasible as it can be completed virtually and is less resource intensive compared to more traditional pharmacist models.

Limitations of our study include the retrospective design which may not account for all potential confounders and does not establish causality, though we did utilize a propensity score matching approach to balance groups on measured confounders. There are approximately 7,600 eligible patients with diabetes aged 18–75 years within our healthcare setting, and this pilot evaluated a smaller sample size for patients who received pharmacist e-consultation. Additionally, we could not evaluate the D5 tobacco-free treatment goal due to a known EHR reporting error. This resulted in some patients meeting D4 at baseline. Less patients met the D4 at baseline in the pharmacist group which was likely due to limited pharmacist e-consult requests for cessation treatment recommendations. We did not include the aspirin goal in our PS matching due to high rates of attainment in the overall population, though fewer patients met this goal at baseline in the pharmacist group. The D5 composite measure is comprised of surrogate outcomes and process measures rather than hard clinical outcomes, such as cardiovascular events, and the long-term impact of pharmacist engagement on patient-centered hard outcomes will need to be determined as this care process continues to be implemented in the practice. Finally, the study was conducted at 13 practices within the same health system. Expansion to other health systems and sies is needed to fully establish the feasibility and effectiveness of the pharmacist e-consultation model embedded within primary care.

Future studies should examine the overall enhanced primary care team model and the pharmacists’ role within it specifically for patients at highest risk for suboptimal diabetes management and diabetes-related complications. This includes racial and ethnic minoritized populations, rural residents, low-income individuals, and patients with limited English proficiency. Further, it will be important to assess the long-term impact of this model on microvascular and macrovascular complications of diabetes, patient satisfaction with care, and clinician burnout.

## Conclusions

The enhanced primary care team model that leverages MMS pharmacists through asynchronous e-consultation is a novel multidisciplinary effort to improve the quality and efficiency of care for patients with diabetes. MMS pharmacists embedded in primary care teams have a high level of expertise in chronic disease management and are often a limited resource. Therefore, it is important that pharmacists continue to find innovative ways to positively impact the care of more patients. This study demonstrates a novel approach for providing this expertise which can supplement more traditional, extensive longitudinal management approaches.
